# Evaluation of masticatory performance and patient satisfaction for conventional and 3D-printed implant overdentures: a randomized crossover study

**DOI:** 10.1186/s12903-024-04389-1

**Published:** 2024-06-08

**Authors:** Mohamed Shady Nabil, Fatma Fathe Mahanna, Mohamed Moustafa Said

**Affiliations:** 1 Removable Prosthodontics Department, Faculty of Dentistry, Mansoura University, Mansoura, 35511 Egypt; 2 Restorative Dentistry and Prosthodontics, College of Dentistry, American University Iraq Baghdad (AUIB), Baghdad, Iraq

**Keywords:** CADCAM overdenture, 3D-printed overdentures, Implant overdenture, Chewing efficiency, Patient satisfaction, Bar overdenture

## Abstract

**Background:**

This crossover clinical study aimed to evaluate and compare masticatory performance and patient satisfaction for patients rehabilitated with conventional heat-cured acrylic resin and 3D-printed mandibular implant overdentures retained with bar attachment.

**Materials and methods:**

Sixteen completely edentulous healthy participants received new conventional dentures. In the mandible, four interforaminal implants were inserted. Following the stage of osseointegration, the bar was constructed in a trapezoidal configuration. Each patient randomly received the following overdentures using a crossover design: (1) conventional heat-cured acrylic resin overdenture and (2) 3D-printed overdenture (developed by scanning of mandibular conventional overdenture). The masticatory performance was assessed by conducting a two-colour mixing ability test at 5, 10, 20, 30, and 50 masticatory cycles. Moreover, the McGill Denture Satisfaction Questionnaire (MDSQ) was employed to assess patient satisfaction. Evaluation was performed after 3 months of using each overdenture. Paired sample t tests were used to compare the masticatory performance and MDSQ scores of patients for both prostheses.

**Results:**

No significant difference in masticatory performance was reported between the two types of overdentures. Regarding patient satisfaction, only the esthetic aspect was significantly better for conventionally processed overdentures than for printed overdentures. Insignificant differences were observed regarding other MDSQ items between the two overdentures.

**Conclusion:**

Within this clinical study, 3D-printed implant overdentures showed promising results in terms of chewing efficiency and patient satisfaction compared to conventionally fabricated implant overdentures.

**Trial registration:**

Retrospectively registered at www.clinicaltrials.gov: NCT06148727.(28/11/2023).

## Background

Supporting removable prostheses with dental implants has been documented to increase masticatory efficiency and maximum bite force. This is presumably due to the improved retention and stability of the implant-supported prostheses [1, 2]. Therefore, rehabilitation with implant-retained/supported overdentures has greatly enhanced retention and masticatory efficiency, decreased pain during mastication and enabled improved utilization of masticatory muscles, enabling patients to consume a variety of foods [3, 4]. 

Implants placed in the anterior mandibular ridge have been used to improve function. A range of attachment systems have been implemented to secure the overdenture, including telescopic crowns, magnetic attachments, ball attachments, and bar attachment systems. The stability and retention of the bar attachment system are superior to those of the other systems [5, 6]. They present a rigid anchorage system between the implant and the overdenture without exerting pressure on soft tissues. This technique involves splinting of implants and distributing stresses caused by masticatory forces across multiple implants [7–9]. Conversely, mucosal hyperplasia beneath the bar may result from inadequate relief, and conventional casting is a time-consuming procedure. Additionally, it could lead to a greater degree of misfit and porosity in the attachment [10, 11]. 

Polymethyl methacrylate (PMMA) has been used in most cases for the fabrication of conventional complete dentures [12]. The increased acceptance of this material by patients can be attributed to its biocompatibility, aesthetic qualities, and simplicity of processing and repair [13]. However, PMMA has a number of drawbacks, including polymerization shrinkage, oral microbial colonization, lack of radio-opacity, the potential for allergic reactions due to residual monomers, deterioration of mechanical properties with time, and poor wear resistance. These problems have introduced the need for novel materials and manufacturing techniques [14, 15]. 

Advancements in digital dentistry have offered new materials and techniques in the fabrication process of this treatment modality. It has invaded the practice of dental work in different fields since its introduction in the 1980s [16]. The initial effort to develop a CAD/CAM system for the production of removable prostheses revealed in 1994 [17]. The advent of digital denture fabrication was initiated in 2012 by Goodacre et al. [18]

There are two primary methods for digitally fabricating removable dental prostheses: additive and subtractive [19]. The subtractive method involves milling the denture base from a prepolymerized resin blank. Subsequently, prefabricated or milled denture teeth are affixed to the milled base. A significant portion of the blank remains underused and is discarded throughout this procedure, which is one of the technique’s drawbacks [20]. The second approach is additive manufacturing (AM), which is alternatively referred to as rapid prototyping (RP) or 3D printing. It entails the implementation of processes to construct three-dimensional models’ layer by layer.

Despite its relatively recent introduction, 3D printing has revolutionized many fields, such as engineering and medicine, including dental work [21]. The utilization of CAD/CAM technology in the fabrication of complete prostheses offers the benefit of simplified laboratory procedures. It has several advantages over conventional techniques, such as dimensional accuracy and standardized fabrication [22]. The aim of this randomized crossover study was to evaluate the impact of the CAD-CAM additive manufacturing technique for implant overdentures on chewing efficiency and patient satisfaction compared to those of conventionally fabricated heat-cured acrylic resin overdentures. The null hypothesis was that there was no significant difference in chewing efficiency or patient satisfaction between the two treatment modalities.

## Materials and methods

### Study design

A randomized crossover design was used. All patients were provided with two different types of prostheses. This design enabled the standardization of result-influencing variables. Additionally, each patient performed as a self-control. Each patient received two prostheses: conventional and 3D-printed implant overdentures. The same operator constructed all the dentures. Randomization was applied to the order of overdenture insertion to reduce the impact of prosthesis use order on the outcomes. Each overdenture was used for three months, followed by two weeks of rest without wearing a denture; The patient then received the alternative variety of overdenture, which was utilized for an additional three months.

### Sample size calculation

The sample size was determined using the findings of a prior clinical trial [23], which revealed a significant difference in chewing efficiency between 3D-printed and conventional prostheses (the α-error was set to 0.05, the β-error was set to 0.20 (80% power), and the effect size was 0.8). The calculated sample size was 12, which was increased to 16 patients to account for any dropouts. The power analysis was performed with the aid of computer software (G*power 3.1.5. Heinrich-Heine-Universität Düsseldorf, Germany).

### Patient selection

**Fig. 1:**
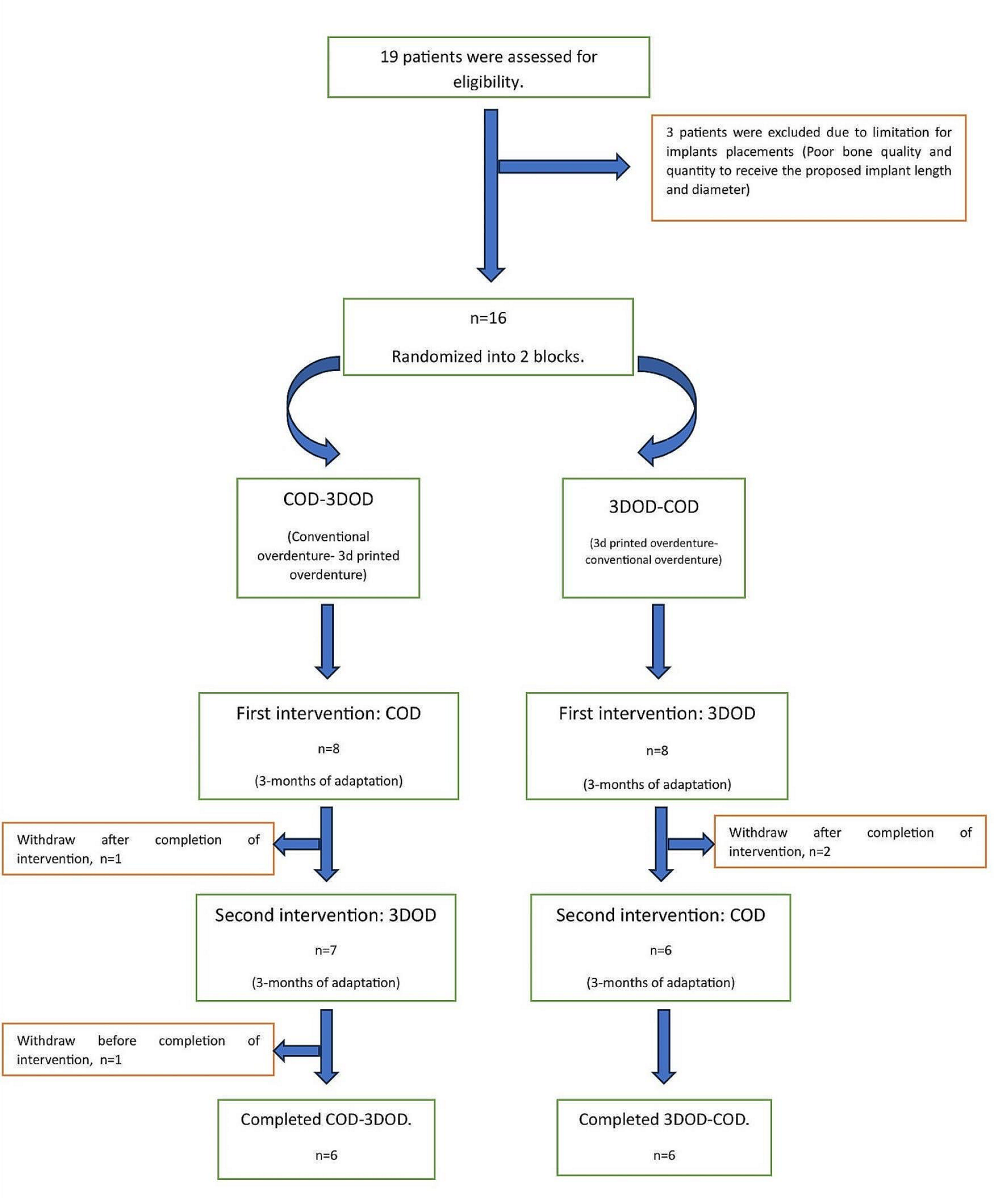
Participant flow diagram. COD: conventional heat-cured acrylic resin overdenture; 3DOD: 3D-printed overdenture

Nineteen participants aged 45 to 60 years (mean 50 years) were screened for this study from the outpatient clinic of the Prosthodontics Department, Faculty of Dentistry, Mansoura University, from January 2021 to August 2021 (Fig. 1). The main complaint for all patients involved in this study was insufficient retention and stability of the mandibular dentures. All patients were enrolled if they met the following inclusion criteria: adequate bone quantity (class IV-VI Cawood and Howell) [24] of the mandible in the lateral incisor and first premolar regions to receive 4 implants in the interforaminal area as verified by preoperative cone beam computerized tomography and adequate restorative space. The following conditions excluded patients from participation: (1) diabetes mellitus and other diseases that affect bone metabolism; (2) smoking, clenching, and bruxism; and (3) neuromuscular and temporomandibular joint disorders. Three patients were excluded due to limitations in implant placement. Sixteen patients were informed about the treatment plan and the need for repeated calling throughout the total period of the study. The patients were fully informed about the purpose and procedures of this study and provided written informed consent. The study was approved by the local ethical committee of the Faculty of Dentistry, Mansoura University (No. A20011122).

### Randomization

Balanced randomization was used to equally assign patients to one of two groups to ensure comparability between groups regarding masticatory efficiency and patient satisfaction. Using random numbers in a Microsoft Excel spreadsheet, participants were randomly assigned to the two groups. The randomization data were generated by a dentist who was blinded to the type of restoration based on the equal distribution of males and females between the two sets. After an adaptation period of three months, masticatory efficiency and patient satisfaction were evaluated for the first eight patients who received conventional overdentures. After a scheduled two-week washout period, the overdenture was replaced with a 3D-printed overdenture, and new recordings were made after an additional three months. The purpose of this random method was to avoid the influence of restoration order on masticatory performance and satisfaction.

### Intervention

**Fig. 2:**
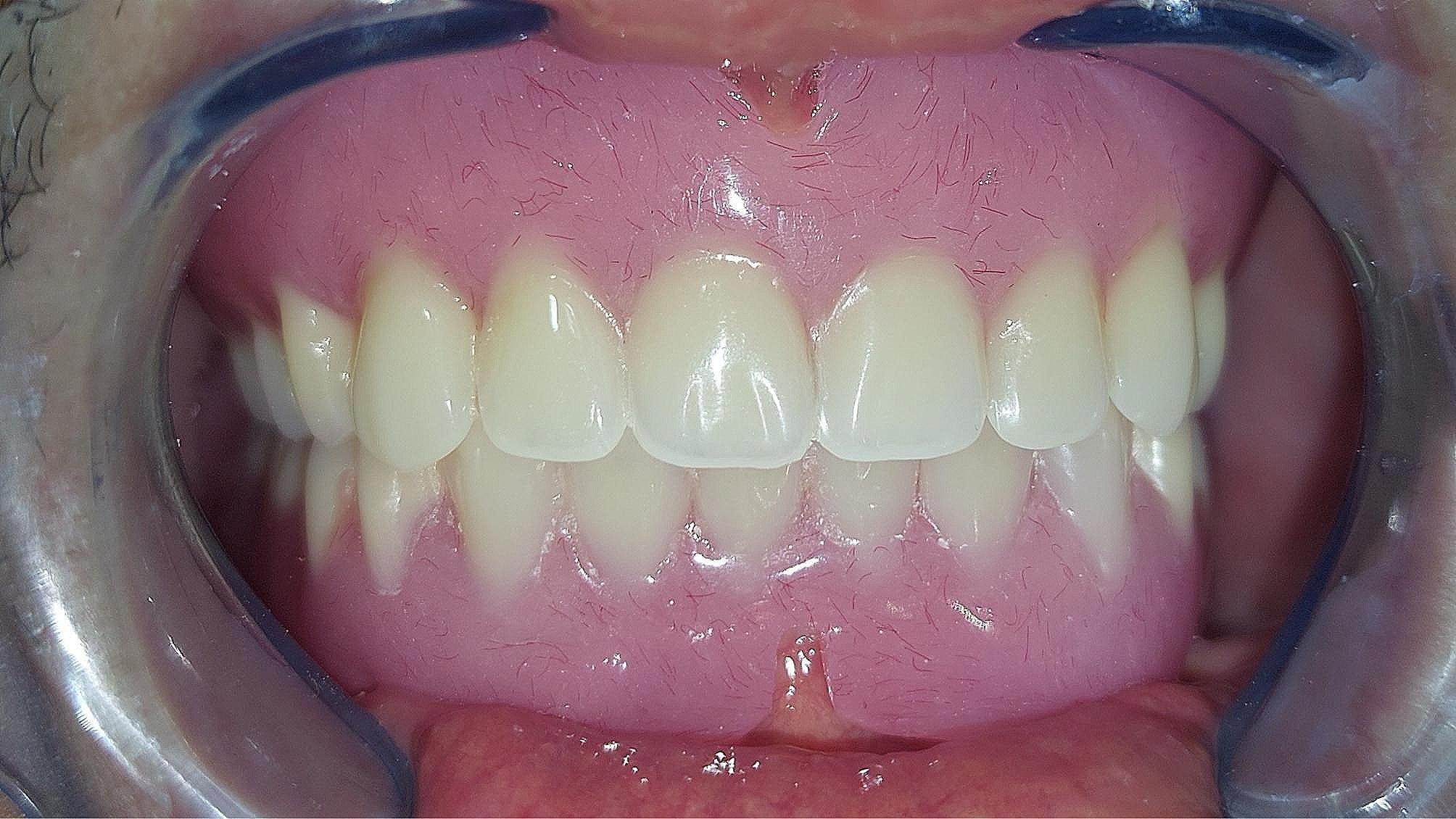
Conventional heat-cured maxillary complete denture and mandibular bar-supported overdenture

For each patient, a new conventional complete denture was constructed. Mandibular dentures were duplicated, gutta-percha markers were inserted in mandibular denture duplicates, and patients were subjected to CBCT. Then, the dentures were scanned alone (dual scan protocol). On the software, both scans were superimposed using gutta-percha markers, and 4 interforaminal implants (Biohorizons trx, Biohorizons, USA) were planned according to the available bone width and length. The position of the fixation screws, and the sleeves was determined, and the plan was used for the construction of the stereolithographic surgical guide. Then, the four implants were placed in their positions using flapless protocol and left for osseointegration. After three months, direct transfer copings were placed, and the copings were splinted using ligature wire and composite resin. Then, a direct transfer impression was made using a perforated custom tray and rubber base impression material (Silagum putty and light, DMG, GmbH, Hamburg, Germany). An impression was poured, and bar was constructed with a trapezoidal configuration. After the bar wax pattern (OT Bar Multiuse - Castable Bar, Rhein83, Italy) was finished, it was tried intraorally and then casted in a Co-Cr alloy. After try-in of casted bar, a bite registration was carried out. Arrangement of lower acrylic semi-anatomic teeth was done then waxing up of dentures to be ready for try in the patient mouth. The bilateral balanced occlusal concept using semi-anatomic artificial composite resin teeth (Bredent, Germany) to increase maxillary denture stability [25, 26]. Flasking of waxed denture and packing of PMMA acrylic resin was done to produce final prosthesis. (Fig. 2) Vents were then made; plastic clips were directly picked up with acrylic resin.

**Fig. 3:**
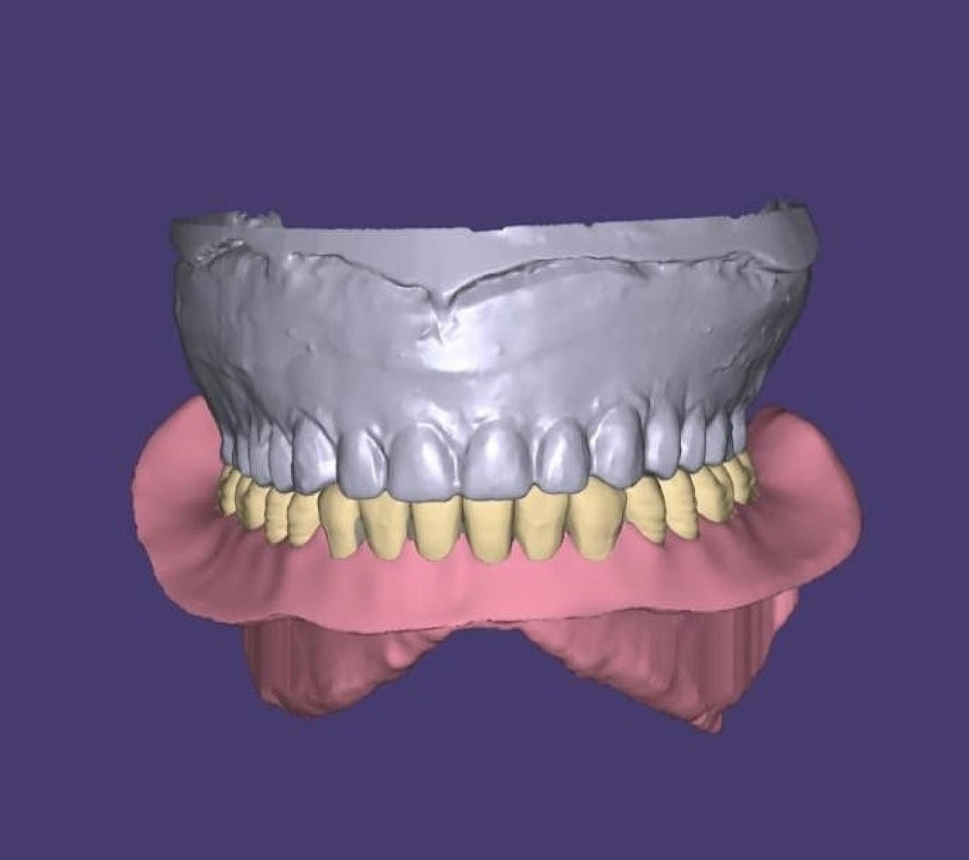
3D image of the designed mandibular overdentures (Exocad)

The master cast/bar assembly of the mandible and the mandibular overdenture were scanned with a 3D scanner (DOF Swing dental scanner, Korea) following a light application of anti-glare spray (Siladent Marmoscan Spray, Basic Ref 250,022 GmbH, Germany) to obtain the standard tessellation language (STL) file format. Using specialized software (Exocad Dental IDB 2.4 plovdiv7290, version 2.4 Engine build 7290, Exocad GmbH) the final complete overdentures were designed over the virtual model. (Fig. 3) Then, the scanned STL image of the conventional overdenture was superimposed onto the newly designed one for comparison of the polished surface, dental alignment, and form of the teeth [27]. 

**Fig. 4:**
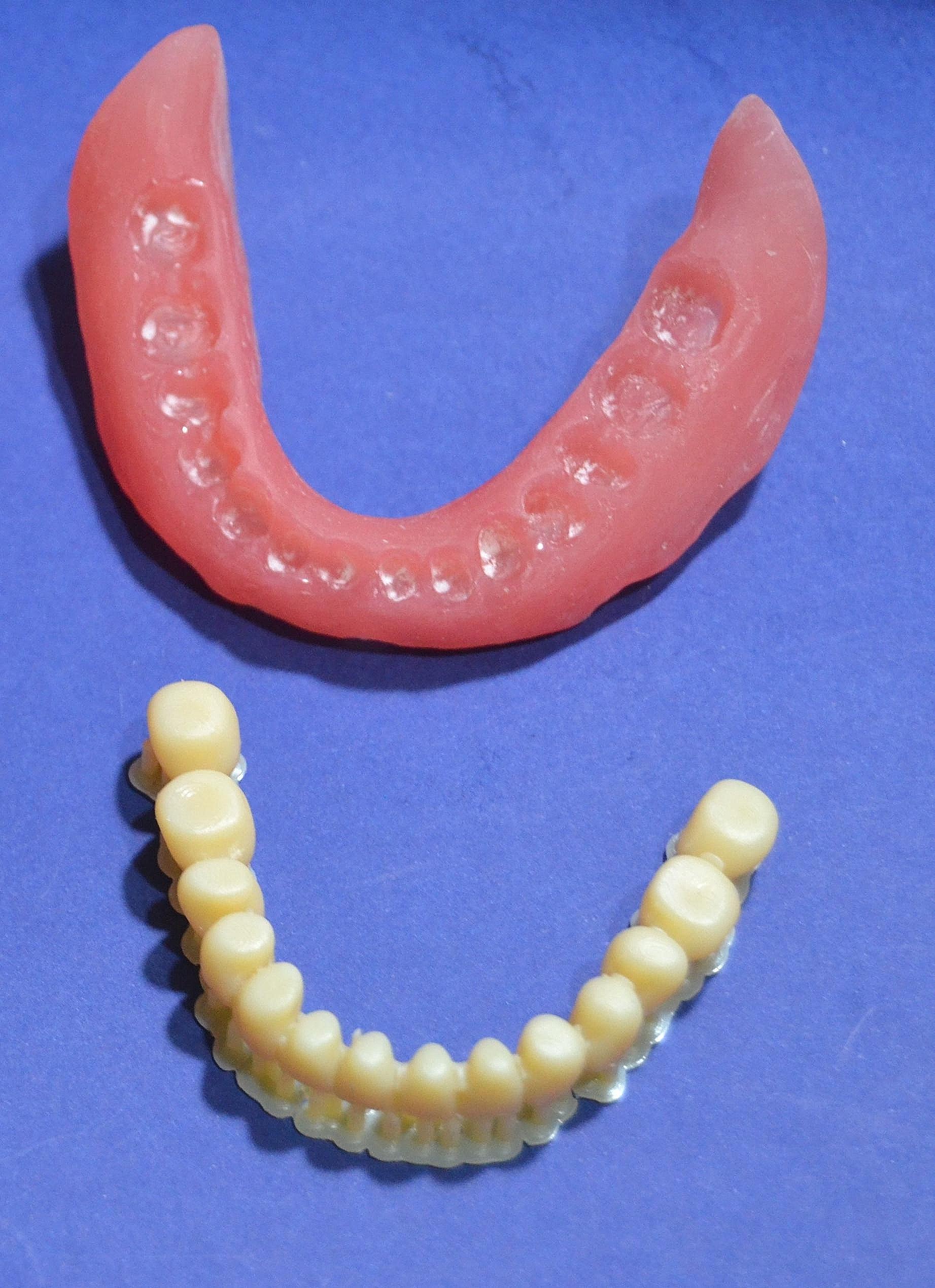
3D-printed denture base and teeth immediately after printing and before bonding

DENTCA Denture Base II (DENTCA, Inc. Torrance, USA), was placed in the cassette of the 3D printer (RASDENT 3D printer, RASPART.eg). The software received the STL file for the denture base model. The denture base was placed on the build platform with its vertical axis. The perimeter of the denture base was created with support and the slice thickness was determined (50 μm), after which the printing process began. The denture teeth were manufactured as a single unit (Fig. 4) using the same technique and tooth resin (DENTCA Denture Tooth, Shade A2, DENTCA, Inc. Torrance, USA).The base was cleansed with isopropyl alcohol (Isopropyl Alcohol Extra Pure, Alpha Chemical) subsequent to the removal of the final supports. The teeth and printed denture base were adhered together using a small quantity of shade-matched light-cured adhesive. Using a traditional technique, wet polishing sand was used to polish the final denture [27]. Then, the plastic clips were picked up to the fitting surface of the 3D-printed denture by the direct functional pickup technique, as performed for conventional overdentures. Four patients dropped out through the follow-up period.

### Evaluation

#### Masticatory performance evaluation

All patients were evaluated for chewing efficiency for both overdentures three months after insertion. Chewing efficiency was evaluated utilizing a two-colour mixing ability test that had been previously documented (colorimetric method) [28] as follows: Chewing gums of two hues (Trident®, Chewing Gum, Mondelez, Egypt) were utilized to create two samples: one flavoured with spearmint (white) and the other with strawberry (red). Two strips of a standardized size (30 × 18 × 3 mm) were manually stuck together. Patients were asked to keep the gum sample intraorally for one minute and then chew it for 5, 10, 20, 30 and 50 strokes, respectively. Five samples were tested with a one-minute interval to reduce fatigue. To make a uniform-thickness wafer, chewed gum was rinsed and sandwiched between two sheets of transparent, rigid plastic with a 1 mm spacer. All samples were then analyzed. From both sides, a Binq 5560c Mirascan® digital scanner (BinQ®, USA) scanned the samples at 600 dots per inch. The scanned image was converted to a fixed size (1175 × 925 pixels) and saved in Adobe Photoshop® format (Photoshop 7.0 ME®—Photo Editor Software—Adobe Systems-Incorporated—USA). Then, the color range tool (fuzziness 20, 25, 30) and histogram function selected the unmixed white areas. From the histogram, both sides’ selected pixels were counted, and their means determined. Subsequently, the mixed fraction (UF) ratio was calculated utilizing the next formula: (Pixels white side a + Pixels white side b) – 2× Pixels of scale/2 × Pixels all. As a reference scale, a scanned piece of unmixed gum was copied in each image (area of 4779 pixelsa) [28]. 

#### McGill denture satisfaction questionnaire

Data on patient satisfaction with their mandibular prosthesis were collected using the eight core items of the McGill Denture Satisfaction Questionnaire (MDSQ) [29]. A single inquiry pertains to the general level of satisfaction with current prosthesis, while the remaining seven inquiries target particular factors that might impact overall satisfaction: comfort, ease of cleaning, speech, aesthetics/ appearance, denture stability, chewing ability, and chewing function. The responses were provided by the participants utilizing a visual analogue scale of 100 mm. Patients were motivated to participate in the survey questionnaire following a three-month period of functioning with each type of prosthesis.

### Statistical analysis

The data were analysed using SPSS (statistical package for social science) computer software (Version 21 SPSS, Chicago. IL, USA). The distribution of the data was tested using the Shapiro–Wilk test of normality. Normally distributed continuous data are described as the mean ± standard deviation. Paired sample t tests were used to compare the masticatory performance and MDSQ scores of patients on two occasions (when using conventional overdentures and when using printed overdentures). P value less than 0.05 indicated a significant difference at the 95% confidence interval.

## Results

A comparison of the masticatory performance evaluation scores between the two groups is shown in Table 1. The hue of variance of the chewing test decreased with increasing number of chewing cycles. In conventional overdenture group, the hue values average was 0.17 ± 0.037, 0.199 ± 0.253, 0.095 ± 0.034, 0.07 ± 0.025 and 0.054 ± 0.022 after five, ten, twenty, thirty and fifty strokes, respectively. In 3D-printed overdenture group, the hue values average was 0.177 ± 0.081, 0.131 ± 0.057, 0.092 ± 0.035, 0.07 ± 0.022, and 0.114 ± 0.248 after five, ten, twenty, thirty and fifty strokes, respectively. There were no statistically significant differences between the two types of overdentures at different chewing strokes (*P* > 0.05). Table 2 shows the mean MDSQ scores for the two groups. Only the esthetics aspect was significantly better in conventionally processed overdentures than in printed overdentures (*P* < 0.05). In contrast, no significant differences were observed for the other MDSQ items between the two groups (*P* > 0.05).

**Table 1:** Mean values of hue variation at different numbers of chewing strokes between conventional and 3D-printed overdentures

	Conventional Mean ± SD	3D-printed Mean ± SD	Paired t-test (*P* value)
Five strokes	0.17 ± 0.037	0.177 ± 0.081	0.813
Ten strokes	0.199 ± 0.253	0.131 ± 0.057	0.405
Twenty strokes	0.095 ± 0.034	0.092 ± 0.035	0.82
Thirty strokes	0.07 ± 0.025	0.07 ± 0.022	0.945
Fifty strokes	0.054 ± 0.022	0.114 ± 0.248	0.409

SD, standard deviation

**Table 2:** MDSQ scores in conventional and 3D-printed overdentures

	Conventional Mean ± SD	3D-printed Mean ± SD	Paired t-test (*P* value)
Satisfaction	62.5 ± 8.1	64 ± 7.5	0.262
Cleaning	69.4 ± 9.4	70.5 ± 8.8	0.570
Speech	65.2 ± 9.1	60.4 ± 8.9	0.772
Comfort	71.2 ± 7.7	70.5 ± 8.4	0.331
Esthetics	74.7 ± 6.7	71.3 ± 7.6	0.011*
Stability	69.3 ± 8.0	69.5 ± 9.0	0.834
Chewing ability	62.6 ± 9.4	62.7 ± 8.7	0.950
Chewing function	58.5 ± 6.5	59.7 ± 7.3	0.191

SD, standard deviation, * significant

## Discussion

This study utilized a within-patient study design that permits the standardization of patient variables. The optimal time to achieve neuromuscular adaptation after the rehabilitation of edentulous patients with implant-supported overdentures remains controversial in the scientific literature. According to Gartner and colleagues [30], one month is sufficient for achieving coordinated muscle activity. Feine and colleagues [31] assessed the masticatory functions of implant-supported fixed and removable mandibular prostheses after a two-month adaptation period. Van Kampen and coworkers [32] demonstrated that three months of implant overdenture rehabilitation is sufficient to achieve good neuromuscular control. Therefore, a 3-month adaptation period was chosen for both overdentures evaluated in this study. The evaluation of chewing efficiency was conducted using scanning and digital assessment of two-colour chewing gum (mixing ability test) because it has several advantages over the sieving method, including a reduction in the time required to process chewed artificial test food samples, cost effectiveness, and ease of application [33, 34]. The MDSQ is a reliable and valid instrument for evaluating the effectiveness of complete dental prostheses in the mandible of edentulous patients [29]. 

At different chewing strokes, there was no statistically significant difference between conventional and 3D-printed implant overdentures. This may be because both overdentures are implant-supported; the support is entirely implant-based. In addition, the occlusal surface was replicated for the 3D-printed overdenture by scanning the conventional denture to standardize the size, alignment, and form of the teeth. The improved masticatory performance of both implant overdentures may be due to the increased retention and stability of the mandibular denture provided by the implants, which reportedly enhanced the patient’s ability to grind food while chewing. Furthermore, dental implants enhance osseoperception [35], improve tactile sensation and stereognosis, and enhance chewing ability [36]. Elsyad and coworkers [25] reported that compared with complete dentures, fixed prostheses and milled-bar overdentures significantly improved masticatory efficiency and biting force. Muller and colleagues [37] observed superior chewing efficiency with implant overdentures and implant-supported fixed prostheses in comparison to a complete denture.

There was no statistically significant difference between the two groups on any of the MDSQ items. Only the aesthetic aspect of conventionally processed overdentures was significantly superior to that of printed overdentures. This may be attributed to differences in the manufacturing technique. Prefabricated artificial teeth have greater visual similarity to real teeth with different visible areas of translucency and optical properties, whereas 3D-printed artificial teeth are similar to resin teeth in terms of physical properties but have a single-colour tone and may become discoloured more readily [38, 39]. There was also a difference in the colour of the pink part of the maxillary denture compared to that of the 3D-printed mandibular overdenture, which might be attributed to patient satisfaction with the aesthetic aspect of the 3D-printed overdenture.

Regarding masticatory performance, both conventional and 3D-printed implant overdentures are reliable and recommended treatment options. In contrast to conventional overdentures, 3D-printed overdentures are anticipated to reduce patients’ physical burden by decreasing the number of clinic visits required for denture fabrication [40, 41]. However, additional research is required to confirm the long-term clinical efficacy of 3D-printed implant overdentures.

This study was limited by its small sample size, short-term evaluation, and washout periods. The implementation of a formal lengthy washout period would have necessitated a long period during which the overdenture was not used; however, it was not ethically permissible to do so in this study. Nonetheless, as demonstrated by the results, the influence of the carryover effect can be considered minimal, as there was no significant difference between the two treatment options regarding masticatory performance.

## Conclusions

Within the limitations of this clinical study, 3D-printed implant overdentures showed promising results regarding chewing efficiency and patient satisfaction compared to conventionally fabricated implant overdentures. 3D-printed implant overdentures could be a viable option for patients with less aesthetic concerns.

## Data Availability

The datasets analysed during the current study are available from the corresponding author on reasonable request.

## Abbreviations

3d: Three dimensional
MDSQ: McGill Denture Satisfaction Questionnaire
CAD/CAM: Computer-aided design and computer-aided manufacturing
PMMA: Polymethyl methacrylate
AM: Additive manufacturing
RP: Rapid prototyping
CBCT: Cone beam computed tomography
Co-Cr alloy: Cobalt-chromium alloy
STL file: Standard tessellation language file format
UV light: Ultraviolet light
UF: Ratio of mixed fraction
